# Disulfiram inhibits neutrophil extracellular trap formation and protects rodents from acute lung injury and SARS-CoV-2 infection

**DOI:** 10.1172/jci.insight.157342

**Published:** 2022-03-08

**Authors:** Jose M. Adrover, Lucia Carrau, Juliane Daßler-Plenker, Yaron Bram, Vasuretha Chandar, Sean Houghton, David Redmond, Joseph R. Merrill, Margaret Shevik, Benjamin R. tenOever, Scott K. Lyons, Robert E. Schwartz, Mikala Egeblad

**Affiliations:** 1 Cold Spring Harbor Laboratory (CSHL), Cold Spring Harbor, New York, USA.; 2 Department of Microbiology, Icahn School of Medicine at Mount Sinai, New York, New York, USA.; 3 Division of Gastroenterology and Hepatology, Department of Medicine, and; 4 Division of Regenerative Medicine, Ansary Stem Cell Institute, Weill Cornell Medicine, New York, New York, USA.; 5 Medical Scientist Training Program, School of Medicine, and; 6 Graduate Program in Pharmacology, Stony Brook University, Stony Brook, New York, USA.; 7 Department of Physiology, Biophysics and Systems Biology, Weill Cornell Medicine, New York, New York, USA.

**Keywords:** COVID-19, Immunology, Innate immunity, Neutrophils

## Abstract

Severe acute lung injury has few treatment options and a high mortality rate. Upon injury, neutrophils infiltrate the lungs and form neutrophil extracellular traps (NETs), damaging the lungs and driving an exacerbated immune response. Unfortunately, no drug preventing NET formation has completed clinical development. Here, we report that disulfiram — an FDA-approved drug for alcohol use disorder — dramatically reduced NETs, increased survival, improved blood oxygenation, and reduced lung edema in a transfusion-related acute lung injury (TRALI) mouse model. We then tested whether disulfiram could confer protection in the context of SARS-CoV-2 infection, as NETs are elevated in patients with severe COVID-19. In SARS-CoV-2–infected golden hamsters, disulfiram reduced NETs and perivascular fibrosis in the lungs, and it downregulated innate immune and complement/coagulation pathways, suggesting that it could be beneficial for patients with COVID-19. In conclusion, an existing FDA-approved drug can block NET formation and improve disease course in 2 rodent models of lung injury for which treatment options are limited.

## Graphical abstract

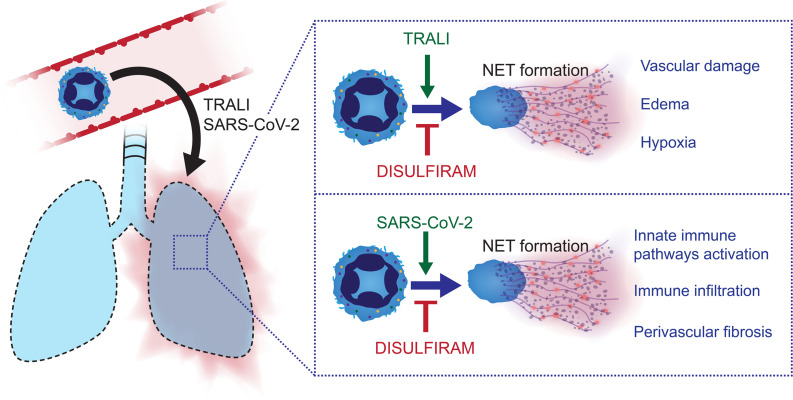

## Introduction

Acute respiratory distress syndrome (ARDS) has a high in-hospital mortality rate that increases significantly with age (1). Excessive formation of neutrophil extracellular traps (NETs) is increasingly being recognized as a key contributor to acute lung injury (ALI) or ARDS (2–4). NETs are web-like extracellular DNA structures that are formed in response to infection and can ensnare and kill oversized (5) or supernumerary pathogens (6). NETs can also be formed in response to tissue damage or viral infections (7–13). NETs are coated with granule-derived proteins, including proteases and histones that are highly cytotoxic (14, 15). Thus, although NET formation can help contain infections, it can also inflict severe damage to the host tissue (4, 14, 16). Indeed, excessive NET formation can directly damage the lung microvasculature (17–20) and promote thrombosis (21–23), leading to multiorgan damage and cardiovascular complications (24). Microvascular damage, thrombosis, and cardiovascular complications are known complications of severe COVID-19 (25). Consistently, elevated levels of NETs are found in the blood, thrombi, and lungs of patients with severe COVID-19 (21, 26–28), suggesting that neutrophils and NETs may play an important role after infection with SARS-CoV-2.

Despite the recognized importance of NETs in a variety of diseases (27), including lung injury and cancer, the only FDA-approved NET-targeting drug is the inhaled drug dornase alfa (recombinant DNase I). DNase I can digest NETs present in the airways once they have formed. However, DNase I does not block NET formation or release, and in its inhaled form, it likely has minimal ability to digest NETs beyond the airways. Blocking NET formation by targeting the signaling pathway leading to NET formation is another approach. The enzyme peptidyl arginine deiminase 4 (PAD4) can citrullinate histones, and this modification is required for NETs to form, though NET formation occurring independently of PAD4 has been reported (e.g., in response to *Candida albicans*; ref. 29). However, while experimental PAD4 inhibitors exist, none has reached clinical trials. The molecule gasdermin D, which has been proposed to form pores in the nuclear and plasma membranes, is important for NET formation (30, 31). Specific gasdermin D inhibitors are in preclinical development, but disulfiram, a drug that has been FDA approved since 1951, was recently shown to inhibit gasdermin D in macrophages and to increase survival after experimental sepsis in mice (32, 33). Here, we tested the ability of disulfiram to inhibit NET formation and to improve disease outcome in a golden hamster model of COVID-19 and in a classical mouse model of ALI (34): transfusion-related ALI (TRALI). Our data suggest that NETs play important roles in COVID-19 pathogenesis, and that disulfiram, a longstanding FDA-approved drug, can efficiently reduce NET formation and lung injury. These findings have immediate therapeutic implications and highlight the importance of the future development of NET inhibitors.

## Results

### Disulfiram, an FDA-approved drug, inhibits NET formation.

**Figure 1:**
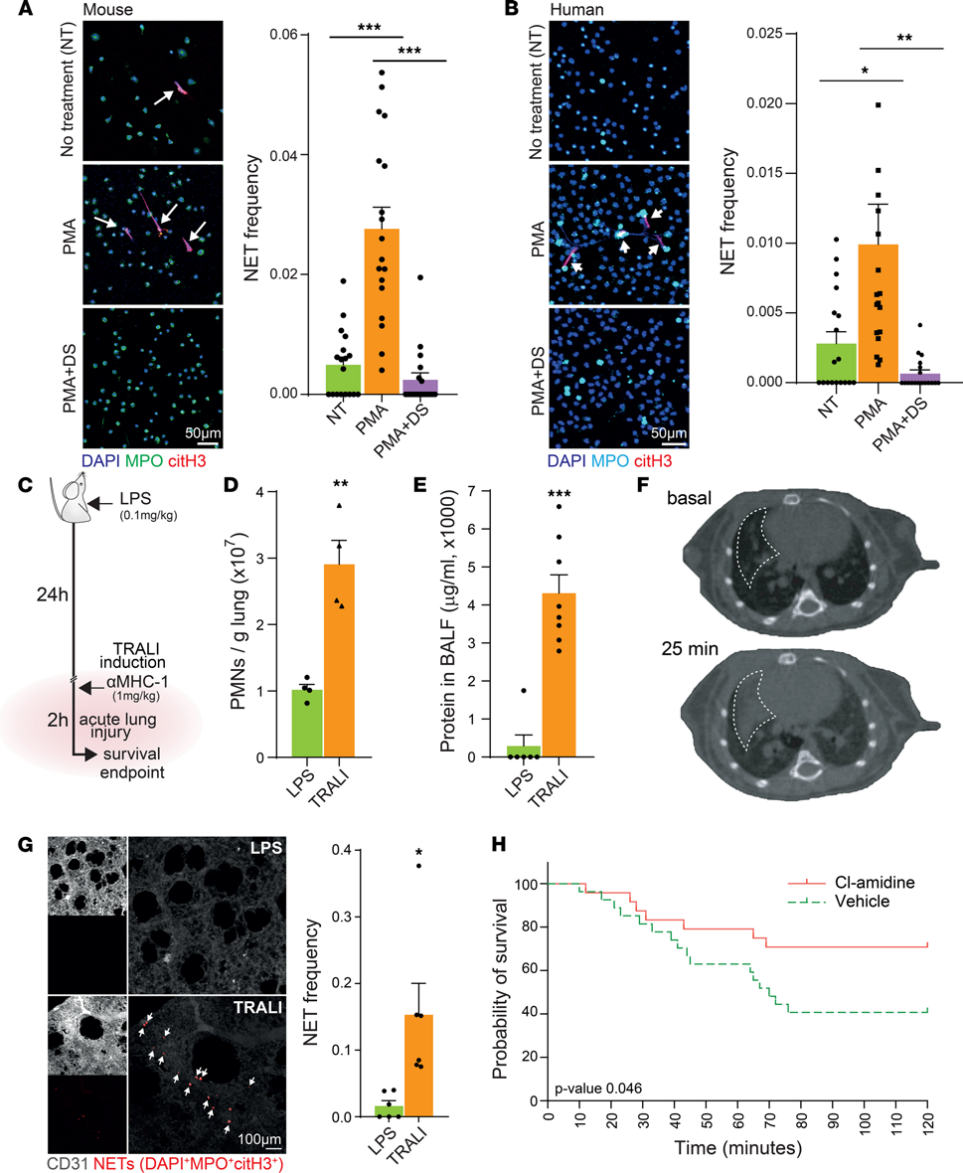
Disulfiram blocks neutrophil extracellular trap (NET) formation, and TRALI is a model of NET-driven lung injury. (**A**) Ex vivo NET formation assay of mouse neutrophils sorted by FACS, unstimulated/untreated (NT) or stimulated with 100 nM of PMA or PMA + 10 μM disulfiram (PMA+DS). NET frequency (NET counts normalized to neutrophil counts, with NETs defined by the triple colocalization events of DNA, myeloperoxidase [MPO], and citrullinated histone H3 [citH3]). *n =* 18 random fields from 4 mice per condition. (**B**) Ex vivo NET formation assay of human neutrophils from RBC-lysed blood, unstimulated or stimulated with PMA or PMA + 10 μM disulfiram (PMA+DS). *n =* 18 random fields from 3 healthy donors per condition. Scale bar: 50 μm. (**C**) Experimental design used to induce TRALI. (**D**) Absolute number of neutrophils (PMNs) infiltrated to the lung upon TRALI, determined by flow cytometry. *n =* 4 mice per group. (**E**) Protein content in the bronchoalveolar lavage fluid (BALF) as a measure of endothelial integrity. *n =* 6 control and 8 TRALI mice. (**F**) Representative longitudinal CT scan of a mouse subjected to TRALI showing edema formation over time (representative of CT scans from 11 independent mice). (**G**) Whole mount tissue clearing images (left, showing CD31 and NETs, defined as the triple colocalization channel of DNA, MPO, and citH3). Quantification (right) of NETs in the lungs of mice 40 minutes after TRALI induction or in mice treated only with LPS. *n =* 6 lungs per group. Scale bar: 100 μm. (**H**) Survival of mice after TRALI induction and treatment with Cl-amidine, a PAD4 inhibitor able to block NET formation, or vehicle. *n =* 27 (vehicle) and 24 (Cl-amidine) mice. Data are shown as mean ± SEM. *P < 0.05, **P < 0.01, ***P < 0.001; as determined by unpaired 2-tailed *t* test analysis (**D**, **E**, and **G**), 1-way ANOVA with Tukey’s multiple comparison test (**A** and **B**), or log-rank (Mantel-Cox) test (**H**). Arrows indicate NETs in **A**, **B**, and **G**.

There is a medical need for drugs that can block the formation of NETs. Disulfiram is FDA approved for the treatment of alcohol use disorder, due to its ability to inhibit aldehyde dehydrogenase (35), but it was recently shown that disulfiram also can block gasdermin D in macrophages (33). Since gasdermin D is important during NET formation, we tested disulfiram’s ability to inhibit NET formation ex vivo (Supplemental Figure 1A; supplemental material available online with this article; https://doi.org/10.1172/jci.insight.157342DS1). Disulfiram efficiently blocked PMA-induced NET formation using neutrophils purified from mouse blood (Figure 1A) or human peripheral blood (Figure 1B), and it blocked NET formation in a dose-dependent manner (Supplemental Figure 1B). Disulfiram also greatly reduced NET formation when using RBC-lysed blood instead of purified neutrophils, an experimental condition that reduces neutrophil manipulation and preserves the possibility of interaction with other blood cell types (Supplemental Figure 1C). Thus, disulfiram blocks NET formation, likely via its known ability to inhibit gasdermin D polymerization (32, 33).

### Disulfiram increases survival in a TRALI model.

To test whether disulfiram blocked NET formation in a NET-driven disease model, we used a 2-step model of TRALI, in which neutrophils (36, 37), platelets (37, 38), and NETs (3, 4) are known to play prominent roles. In this model, mice are first injected with a low dose of LPS, and then 24 hours later, they are injected with antibodies against major histocompatibility complex I (MHC-I) (Figure 1C). Within minutes of the antibody injection, severe and acute lung failure develops. Mice subjected to this protocol exhibit acute infiltration of neutrophils to the lung (Figure 1D and Supplemental Figure 1D), vascular damage leading to the leakage of plasma proteins to the alveolar space (Figure 1E), and edema in the lungs (Figure 1F). As previously reported (3, 4, 38), we found that neutrophils formed NETs in the lungs of mice subjected to TRALI (Figure 1G) and that treatment with the PAD4 inhibitor Cl-amidine (3, 39, 40), which blocks PMA-induced NET formation (Supplemental Figure 1E), increased overall survival (Figure 1H).

**Figure 2:**
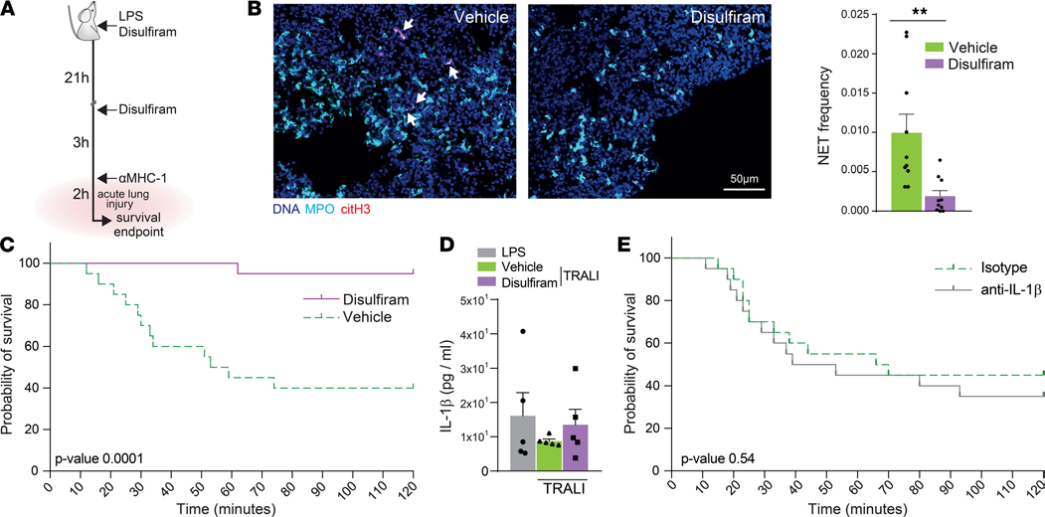
Disulfiram blocks NET formation in vivo and protects against acute lung injury. (**A**) Experimental design. (**B**) Whole mount tissue clearing images (left) and quantification (right) of NETs formed in vivo upon TRALI induction in mice treated with disulfiram or vehicle. *n =* 10 lung volumes from 7 mice per group. Scale bar: 50 μm. Arrows point to NETs. (**C**) Survival curve of mice treated with 50 mg/kg disulfiram in sesame oil 24 hours and 3 hours before TRALI induction. *n =* 20 mice per group. (**D**) IL-1β measurement in lung lysates of LPS-only–treated control mice or mice subject to TRALI induction and treated with vehicle or disulfiram. *n =* 5 mice per group, lungs acquired 40 minutes after TRALI induction. (**E**) Survival curves of mice treated i.v. with 50 μg of IL-1β blocking antibodies or isotype control antibodies 5 minutes prior to TRALI induction. *n =* 20 mice per group. Data are shown as mean ± SEM. **P < 0.01, as determined by 1-way ANOVA with Tukey’s multiple comparison test (**D**) or unpaired 2-tailed Student’s *t* test (**B**). Survival plots show the probability of survival as determined by log-rank (Mantel-Cox) test (**C** and **E**).

To determine whether disulfiram could inhibit NET formation in vivo in the TRALI model, we treated mice with 50 mg/kg of disulfiram i.p. 24 hours and 3 hours before injection with antibodies against MHC-I (hereafter termed TRALI induction; Figure 2A). Disulfiram treatment reduced the number of NETs found in the lungs compared with vehicle-treated mice (determined from whole mount tissue cleared lungs; Figure 2B and Supplemental Videos 1 and 2), without affecting the percentage of neutrophils of all WBCs or the absolute number of neutrophils in the circulation (Supplemental Figure 2, A and B). Neutrophil and monocyte infiltration to the lungs was also unaffected by disulfiram treatment (Supplemental Figure 2, C–F). Importantly, disulfiram treatment caused a dramatic increase in survival: from 40% to 95% (Figure 2C; *n =* 20 mice per group, *P =* 0.0001). Since gasdermin D blockade in macrophages can reduce IL-1β release (33), we examined whether disulfiram might, in part, increase survival in the TRALI model by targeting IL-1β secretion. However, we found no increase in IL-1β protein levels in the lungs of mice after TRALI induction, regardless of whether or not mice were treated with disulfiram (Figure 2D). Furthermore, treatment with anti–IL-1β antibodies (50 μg i.v. injected 5 minutes prior to TRALI induction, a dosage reported to block IL-1β in mice; ref. 41) did not increase survival compared with treatment with isotype control antibodies (Figure 2E). Hence, the ability of disulfiram to increase survival in the TRALI model correlated with its ability to reduce NET levels in the lung, but not with changes in IL-1β levels.

We next tested other approaches to target NETs in the context of ALI. Inhaled DNase I is FDA approved and used in cystic fibrosis where NETs accumulate in the alveolar space, increase mucus viscosity, and impair gas exchange (42, 43). When we administered 200 U of DNase I intranasally (i.n.), 5 minutes prior to the induction of TRALI, no increase in survival was obtained over vehicle (Supplemental Figure 2G). Neutrophil-platelet interactions are important in models of ALI and can induce NET formation (38). Additionally, NETs and platelets form a forward-feedback loop that can drive the formation of thrombi (23, 44, 45), possibly also in severe COVID-19 (46–49). However, disruption of neutrophil-platelet interactions (50, 51) by administering the glycoprotein IIb/IIIa inhibitor tirofiban (Supplemental Figure 2H) 1 hour before TRALI induction or the phosphodiesterase 3 (PDE3) inhibitor dipyridamole (Supplemental Figure 2I) 24 hours and 3 hours before TRALI induction resulted in a small or no survival benefit, respectively. Taken together, these data suggest that systemic inhibition of gasdermin D and NET formation in vivo using disulfiram, an existing FDA-approved drug, shows significant survival benefits in a mouse model of TRALI, as compared with multiple other approaches to target NETs.

### Disulfiram improves lung function upon TRALI.

**Figure 3:**
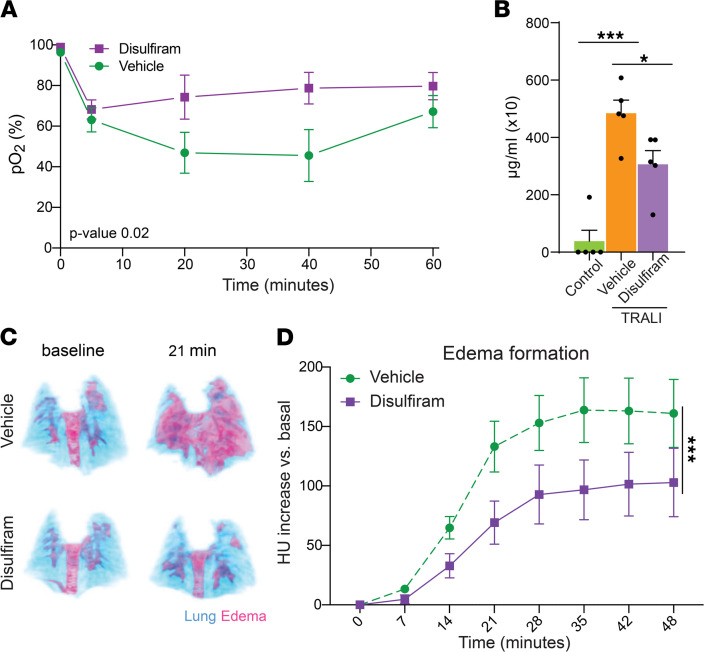
Disulfiram treatment improves key respiratory parameters upon TRALI induction. (**A**) pO_2_ measured longitudinally on surviving mice after TRALI induction and treatment with disulfiram or vehicle. *n =* 4 (vehicle) and 3 (disulfiram) mice. (**B**) Protein content in the BALF of naive mice or mice after TRALI induction and treatment with either disulfiram or vehicle. *n =* 5 mice per condition. (**C**) Representative projections from longitudinal CT scans of mice after TRALI induction and treatment with disulfiram or vehicle, showing the lung volume (in blue) and water-dense tissue (edema, in red). Representative of *n =* 10 mice per group. (**D**) Quantification of the longitudinal CT scans of mice after TRALI induction and treatment with disulfiram or vehicle. Basal HU units (prior to TRALI induction) were subtracted from all subsequent measurements to represent the increase in edema formation. *n =* 10 mice per group. Data are shown as mean ± SEM. *P < 0.05, ***P < 0.001, as determined by 1-way ANOVA with Tukey’s multiple comparison test (**B**) or 2-way ANOVA (**A** and **D**).

After determining that disulfiram blocked NET formation and improved survival of mice upon TRALI induction, we next set out to determine the treatment’s effect on lung function. Upon TRALI induction, breathing rate sharply decreased but disulfiram stabilized it compared with the vehicle-treated controls, which continued to experience a further decline in breath rate (Supplemental Figure 3A). Disulfiram also resulted in a striking improvement of partial pressure of oxygen (pO_2_) after TRALI induction compared with vehicle-treated survivors: a reduction in pO_2_ was observed during the first 10 minutes after TRALI induction for both disulfiram- and vehicle-treated mice; however, at the 20- and 40-minute time points, the disulfiram-treated mice showed greatly improved oxygenation, whereas the surviving vehicle-treated mice did not show improvement until the 60-minute time point (Figure 3A). We excluded the nonsurviving vehicle-treated mice from this analysis, since all the disulfiram-treated mice in the experiment survived, but we note that oxygenation for the nonsurviving vehicle-treated mice never improved before they succumbed to the lung injury (Supplemental Figure 3B). These data suggest that disulfiram does not protect against the initial lung damage but prevents the further progression, as observed in vehicle-treated animals. Consistent with this idea, we found that, although disulfiram significantly reduced the protein content of bronchoalveolar lavage fluid (BALF; Figure 3B), the levels were still elevated compared with control mice without TRALI induction. To directly analyze the kinetics of edema formation in disulfiram- and vehicle-treated mice during TRALI, we used longitudinal CT (Supplemental Figure 3C). In these experiments, we found a stark reduction in the volume of edema in the lungs of disulfiram-treated mice compared with their vehicle-treated littermates (Figure 3, C and D; Supplemental Figure 3D; and Supplemental Video 3). Taken together, these data suggest that disulfiram treatment did not affect the initial lung damage after TRALI induction, but it reduced the progressive vascular damage and edema accumulation, thereby improving oxygenation. It is likely these effects are responsible for the strikingly increased survival of disulfiram-treated mice upon TRALI induction.

### Disulfiram treatment reduces the gene expression of pathways regulating innate immunology and coagulation after SARS-CoV-2 infection in rodents.

**Figure 4:**
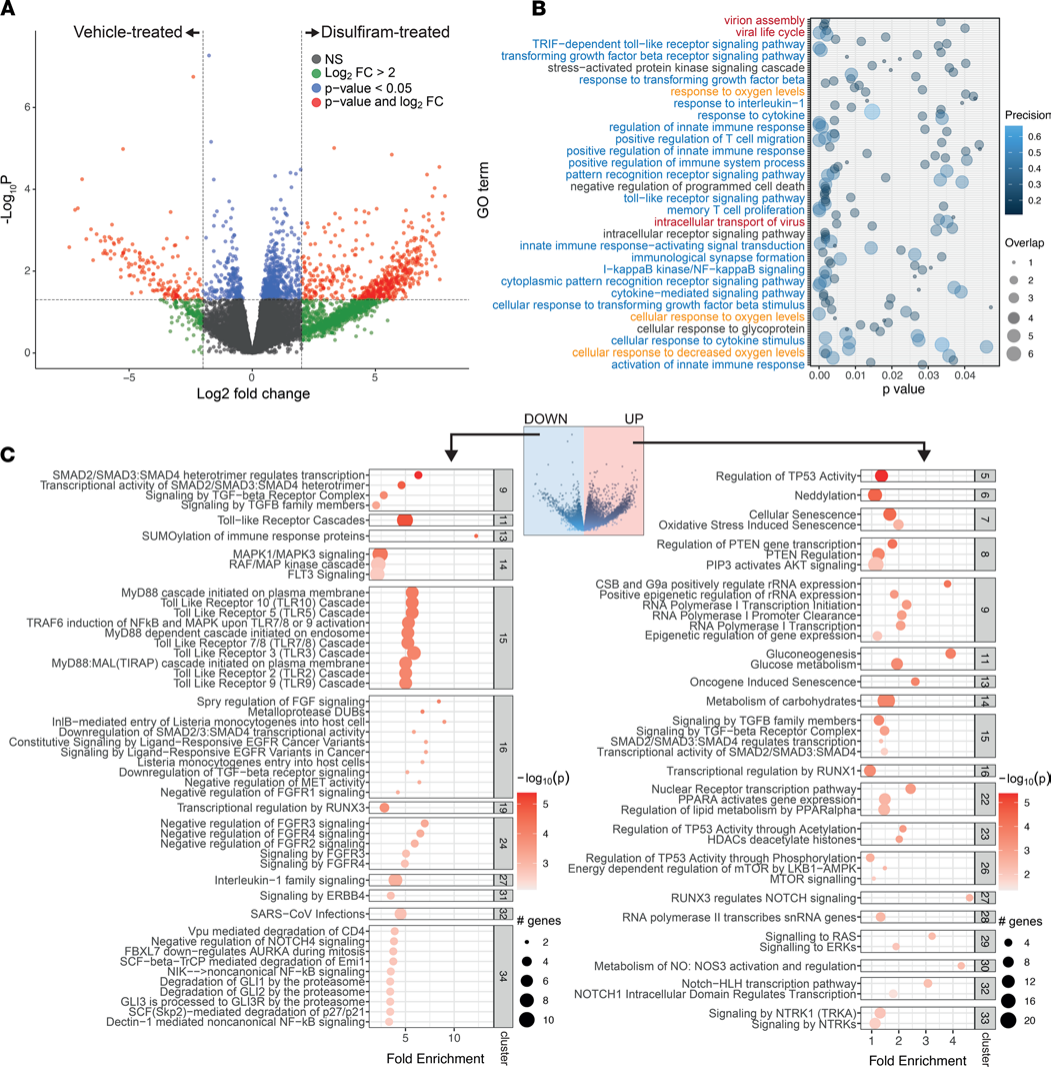
RNA-Seq data from lungs of infected hamsters treated with disulfiram or vehicle. (**A**) Volcano plot of log_2_FC (log_2_ of the fold change) versus –log_10_*P* (log_10_ of the *P* value) of all genes in the data set. Positive and negative values on the *x* axis represent genes upregulated and downregulated, respectively, by disulfiram treatment. Green dots show genes with a log_2_ fold change over 2 between conditions, blue dots represent genes with a *P* value under 0.05, and red dots show those genes that have both a log_2_ fold change > 2 and *P* < 0.05. (**B**) GO biological processes terms enriched in the whole differentially expressed genes list highlighting some of the terms (full list in Supplemental Table 2) related to immune functions (blue), response to oxygen levels (orange), other (black), and viral life cycle (red). (**C**) Clustering of Reactome pathways enriched in the genes downregulated (left) or upregulated (right) in response to disulfiram in SARS-CoV-2–infected golden hamsters. Some of the clusters (gray squares) are shown here (full list in Supplemental Table 4). Color and bubble size reflect the –log_10_ of the *P* value for that pathway and the number of genes present in the data set belonging to a particular pathway, respectively.

TRALI is a rare type of lung injury; however, lung injury is a major medical problem in the ongoing COVID-19 pandemic. To test whether disulfiram could be useful in treating COVID-19, we used a golden hamster model (52) and infected hamsters by i.n. injection with 1 × 10^3^ plaque-forming units (PFUs) of SARS-CoV-2. We evaluated the effects of disulfiram treatment by performing RNA-Seq of lung tissues from SARS-CoV-2–infected hamsters 6 days after infection. We compared vehicle-treated and disulfiram-treated hamsters (with disulfiram treatment starting either 1 day before or 1 day after infection). Disulfiram treatment led to profound alterations in gene expression with more than 1000 differentially expressed genes (*P <* 0.05; Figure 4A, Supplemental Figure 4A, and Supplemental Table 1). The Gene Ontology (GO) terms showed that the genes with altered expression belonged to several molecular functions and biological pathways (Figure 4B, and Supplemental Table 2). Among the GO terms regulated upon disulfiram treatment were several related to innate immune function (such as response to IL-1, Pattern Recognition Receptor [PRR], and TLR signaling or cytokine signaling), response to oxygen levels, and response to viral life cycle (Figure 4B; blue, orange, and red text, respectively).

We next analyzed the pathways represented by the up- and downregulated genes after disulfiram treatment. While the pathways of the upregulated genes were not particularly revealing (Supplemental Figure 4B), the pathways associated with the genes downregulated by the disulfiram treatment included many related to the innate immune response (Supplemental Figure 4C and Supplemental Table 3), including TLR cascades and IL-1 signaling. These data suggest that disulfiram treatment may help control an exacerbated innate immune response against SARS-CoV-2. Of note, IFNs are key for antiviral immunity, including against SARS-CoV-2 (53, 54), and we found increased IFN signaling genes and IFN regulatory factors in the disulfiram-treated lungs compared with the vehicle-treated controls (Supplemental Figure 4, D and E), indicating that IFN responses are not reduced by disulfiram-mediated NET inhibition.

Next, we performed clustering of the Reactome pathways of the up- and downregulated genes, a method that allows a more in-depth analysis by aggregating biologically related terms together (55). Interestingly, among the clusters obtained from the disulfiram downregulated genes, we found not only the aforementioned immunity pathways (i.e., in clusters 11, 13, 14, 15, 27, and 34), but also a SARS-CoV-2 infection pathway (cluster 32) (Figure 4C, Supplemental Figure 4F, and Supplemental Table 4). These data indicate that at least some members of the infection pathway are downregulated upon disulfiram treatment. Genes that were upregulated in response to disulfiram treatment were associated with several transcriptional pathways (i.e., clusters 9 and 28), nitric oxide production (e.g., cluster 30), metabolic pathways (e.g., clusters 11, 14, or 22), and also P53-related pathways (e.g., cluster 5, 23, or 26) (Figure 4C and Supplemental Table 5).

Finally, when we interrogated the KEGG pathways (Supplemental Figure 4G), we found enriched complement and coagulation cascades (cluster 11) in the genes downregulated in the disulfiram-treated group, as well as a cluster including vasopressin-regulated pathways (cluster 10). Activation of both the coagulation cascades and vasopressin is associated with adverse clinical outcomes in COVID-19 patients (49, 56). Among the genes upregulated in disulfiram-treated lungs, we found enrichment of the oxytocin pathways (cluster 11), which has been suggested to be protective against SARS-CoV-2 (57). These data indicate that disulfiram treatment altered the immune response of the SARS-CoV-2–infected lung. Of note, similar functional annotation profiles were achieved regardless of whether the daily disulfiram treatment was started preventatively or 24 hours after infection (see Supplemental Tables 6–8 for the list of differentially expressed genes, Reactome pathways, and GO terms of hamsters treated 24 hours after infection; see Supplemental Table 9 for common GO terms between pre- and posttreatment). Taken together, our RNA-Seq data suggest that disulfiram dampens the exacerbated innate immune response after SARS-CoV-2 infection, without impairing natural immunological control of the virus.

### Disulfiram reduces NET formation and improves lung histology in SARS-CoV-2–infected golden hamsters.

**Figure 5:**
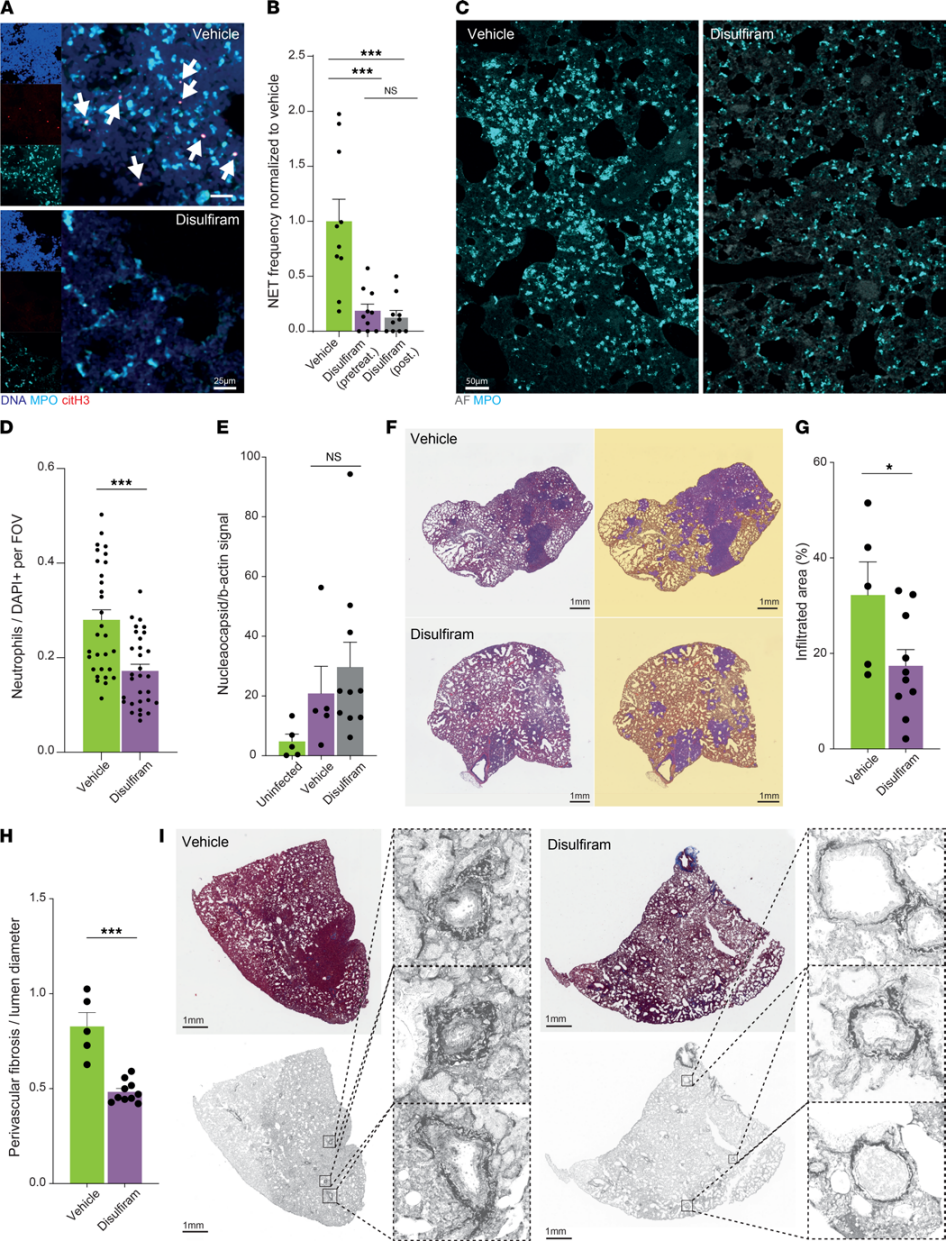
Disulfiram improves lung histology in a golden hamster SARS-CoV-2 infection model. (**A**) Representative images from whole mount cleared SARS-CoV-2–infected lungs from hamsters treated with disulfiram or vehicle. Arrows point to NETs, defined as triple colocalization events of DNA, MPO, and citH3. Representative of 5 independent whole mounts per group. (**B**) Quantification of NETs in the lungs of SARS-CoV-2–infected hamsters. A group was started on daily disulfiram treatment 24 hours prior to infection (pretreat.), while disulfiram was initiated in the other group one day post infection (post.). *n =* 10 lung volumes from 5 hamsters per group. (**C** and **D**) Representative images (showing MPO in cyan) and quantification of neutrophil infiltration to the lungs of SARS-CoV-2–infected hamsters. *n =* 30 random fields from 5 lungs per group. (**E**) Quantification of SARS-CoV-2 nucleocapsid signal normalized to β-actin (both proteins detected in lung lysates by Western blot) in disulfiram- and vehicle-treated hamsters, showing that disulfiram does not affect viral load. *n =* 5 Western blots from 3 uninfected hamsters, 5 Western blots from 5 infected and vehicle-treated hamsters, and 10 Western blots from infected and disulfiram-treated hamsters (5 from the pretreatment and 5 from the posttreatment groups). (**F** and **G**) Representative images (left, original image; right, detection overlay showing infiltrated area in violet) and quantification of the heavily immune-infiltrated areas from H&E-stained lungs of disulfiram- or vehicle-treated hamsters infected with SARS-CoV-2. *n =* 5 (vehicle) and 10 (disulfiram) lungs per group. (**H** and **I**) Quantification (mean value of 10 independent measurements per lung) and representative images of perivascular fibrosis in the Masson trichrome–stained lungs of infected hamsters treated with disulfiram or vehicle. *n =* 5 (vehicle) and 10 (disulfiram) lungs per group. Data are shown as mean ± SEM. *P < 0.05, ***P < 0.001 as determined by 1-way ANOVA with Tukey’s multiple comparison test (**A**) or unpaired 2-tailed *t* test analysis (**D**, **E**, **G**, and **H**). Scale bars: (**A**) 25 μm, (**C**) 50 μm, (**F** and **I**) 1 mm.

In the TRALI model, disulfiram treatment reduced NET formation, so we next tested whether it had similar effect after SARS-CoV-2 infection in hamsters. SARS-CoV-2 infection induced NET formation in the lungs of the infected hamsters (Supplemental Figure 5A), and disulfiram treatment significantly reduced the formation of NETs (Figure 5, A and B, and Supplemental Videos 4 and 5). Since comparable reductions in NET formation were observed whether disulfiram treatment started before or after SARS-CoV-2 infection, the 2 treatment groups were pooled for subsequent analyses. In addition to reduced NET formation, we found reduced neutrophil infiltration in the lungs of the disulfiram-treated hamsters compared with those of vehicle-treated hamsters (Figure 5, C and D), consistent with the previously reported chemoattractant effects of NETs (23) and our RNA-Seq data results showing reduced innate immune function. Thus, both the number of neutrophils and the percentage of neutrophils forming NETs were reduced in the lungs after disulfiram treatment, together greatly reducing the total number of NETs. In contrast, the viral load (as determined by the amount of nucleocapsid protein present in the lungs of the infected hamsters) was unaffected by disulfiram treatment (Figure 5E and Supplemental Figure 5B), indicating that disulfiram treatment does not impair viral clearance. Together with the RNA-Seq analysis and the analysis of NET formation, this suggests that disulfiram treatment affects the host response to the infection but not the SARS-CoV-2 life cycle or viral clearance.

Golden hamsters do not succumb to SARS-CoV-2 infection, so the response to disulfiram treatment was evaluated at the histological level from H&E- and Masson trichrome–stained lung sections. We found that disulfiram treatment was associated with a significant reduction of heavily infiltrated lung area (determined using pixel classifiers that detect hematoxylin-rich areas) (Figure 5, F and G) and with a trend toward reduced total number of cells (number of cells per total lung area, excluding the alveolar spaces) in the lungs of disulfiram-treated hamsters (Supplemental Figure 5, C and D). There was also a stark reduction of perivascular fibrosis in the lungs of disulfiram-treated hamsters versus those of the vehicle-treated hamsters (Figure 5, H and I). Perivascular fibrosis is associated with increased perivascular edema (58), and consistently, there was a trend toward increased open alveolar space in the lungs of the disulfiram-treated hamsters versus those of the vehicle-treated hamsters (Supplemental Figure 5E). Thus, in golden hamsters infected with SARS-CoV-2, disulfiram treatment reduces NET formation, as well as lung inflammation and perivascular fibrosis. This suggests that NET formation contributes to the lung damage during SARS-CoV-2 infection. In contrasts to treatment with disulfiram, treatment with dexamethasone, which has been widely used in the treatment of hospitalized COVID-19 patients (59), did not significantly improve the open alveolar space or the number of cells per lung area in the SARS-CoV-2–infected hamsters (Supplemental Figure 5, F–H), but it did significantly decrease perivascular fibrosis (Supplemental Figure 5I). Dexamethasone treatment also did not affect the extent of heavily infiltrated lung areas (Supplemental Figure 5, J and K) or neutrophil infiltration (Supplemental Figure 5, L and M). Additionally, the viral load was actually increased in the lungs of dexamethasone-treated hamsters, consistent with its ability to dampen immune responses, whereas it was not altered by disulfiram treatment (Supplemental Figure 5N). Neither disulfiram nor dexamethasone significantly altered the weight loss observed after SARS-CoV-2 infection (Supplemental Figure 5O). Taken together, these data suggest that, in SARS-CoV-2 infected golden hamsters, disulfiram treatment confers a benefit and works through a different mechanism of action than dexamethasone.

## Discussion

Several diseases have been linked to excessive NET formation, including ARDS and cancer (60). NETs may also play a role in the pathology of severe COVID-19 (e.g., as a cause of lung damage and immune thrombosis) (21). Here, we show that the blockage of NET formation by the FDA-approved drug disulfiram is associated with dramatically improved survival in a model of TRALI and with improved lung histology in a model of COVID-19. The latter results support the notion that NETs may be drivers of severe COVID-19 pathology (17, 18, 22, 27, 28), although we cannot attribute the effect of disulfiram solely to its ability to block NETs, as the treatment caused a global reduction in activation of innate immune signaling pathways. Disulfiram has been used since 1951 and has a well-understood and generally manageable side effect profile (61). Similar to our study on lung injury, a recent report showed a correlation between disulfiram’s ability to reduce NET formation and improved survival in a sepsis mouse model (62). Together with our results, this suggests that disulfiram could be useful in the management of pathologies involving NETs, including lung injuries (23, 60, 63, 64), sepsis (62), thrombosis (65), and cancer (66–69). Disulfiram is not compatible with alcohol consumption due to its ability to inhibit aldehyde dehydrogenase (35). Nevertheless, its strong inhibitory effect on NET formation and its improvement of disease outcomes in multiple rodent models highlight the potential for the future development of safe and effective inhibitors of NET formation.

Disulfiram blocked NET formation efficiently in every setting we tested, whether ex vivo or in vivo and whether targeting human, mouse, or golden hamster neutrophils. Reducing NETs in the mouse TRALI model using DNase I or a PAD4 inhibitor is associated with increased survival, as well as reduced endothelial damage and edema formation (3). Importantly, disulfiram treatment recapitulated these benefits. Disulfiram showed by far the best effects compared with other pharmacological approaches for inhibiting NET formation, including DNase I or PAD4 inhibitors. However, mortality in the TRALI model occurs within minutes, which is much faster than in, for example, COVID-19, and we acknowledge that drugs that showed limited benefit in the TRALI model could still offer protection in other NET-driven diseases. When comparing treatment approaches, we also notice that the administration route of the drugs was different: since DNase I is only available for human use in inhaled form, we administered it via the intranasal route. However, although DNase I can improve symptom management in cystic fibrosis where NETs are found in the alveolar space (42, 43), inhaled DNase I had limited effect on survival in the TRALI model, where intravascular NETs have been reported (3). Thus, we note that the intranasal route may not deliver sufficient levels of drugs to the location where NETs need to be targeted during ALI. A separate issue is that DNase I targets NETs that have already formed and have already been released, while disulfiram and PAD4 inhibitors prevent the formation of NETs from the beginning. DNase I degradation of the DNA backbone of NETs has been reported to leave several other components of NETs in situ, including proteases and histones (70), and these may be detrimental in certain contexts (71). It is also unclear whether the release of NET-entrapped cytotoxic compounds into the bloodstream would have a detrimental effect elsewhere. Hence, drugs that block NET formation and those that digest them may affect disease progression differently and may also have different side effects.

An interesting difference between the TRALI and SARS-CoV-2 models was that disulfiram reduced neutrophil infiltration in the latter but not the former model. NETs expose cytoplasmic material and can increase neutrophil infiltration through a variety of mechanisms (72). We speculate that, during the short time span of the TRALI model (2 hours), there is not enough time to observe a NET-driven, secondary neutrophil infiltration. Additionally, disulfiram does not appear to inhibit the primary infiltration of neutrophils observed after injection of anti–MHC-I antibodies. In contrast, in the SARS-CoV-2 model, the disease develops over the course of several days, allowing sufficient time for NETs to drive secondary neutrophil infiltration, an effect that disulfiram would be able to block by preventing NET formation. We therefore interpret the difference in the effects of disulfiram on neutrophil infiltration to derive, at least partially, from the ability of disulfiram to reduce neutrophil infiltration secondary to NET release, an effect that would only be observed in the SARS-CoV-2 model.

The effect of disulfiram treatment on the survival of mice experiencing TRALI was very dramatic and correlated with reduced NET formation in the lungs, consistent with the hypothesis that it acts by blocking gasdermin D in neutrophils. Gasdermin D is also required for pyroptosis and is involved in IL-1β secretion (73), and disulfiram has been shown to block these processes (32). In the TRALI model, our data suggest that IL-1β plays a minimal role, as (a) IL-1β levels in the lungs were not increased upon TRALI induction, and (b) treatment with IL-1β blocking antibodies did not increase survival. Nevertheless, increased IL-1β secretion likely contributes to COVID-19 (74), though blocking antibodies did not reduce mortality in clinical trials of severe COVID-19 (75). While we focused on the effect of disulfiram in NET-formation, we cannot exclude that disulfiram may also improve disease outcome by affecting other processes, such as pyroptosis and IL-1β release — especially in the SARS-CoV-2 infection model. Experimentally untangling the effects of blocking gasdermin D in neutrophils versus, for example, macrophages would require the availability of conditional KO models. We did observe that the IL-1β pathway was enriched among the significantly downregulated genes upon disulfiram treatment on SARS-CoV-2–infected hamsters, which would be consistent with inhibition of gasdermin D also in macrophages. Thus, it is possible that disulfiram could benefit patients by reducing both IL-1β secretion and NET formation. Of note, disulfiram appeared in a high-throughput screening for drugs able to inhibit M^pro^, a SARS-CoV-2 protease, implying that, in the context of COVID-19, disulfiram may confer protection beyond host effects (76).

The RNA-Seq analysis showed that many genes downregulated by disulfiram belonged to pathways related to the innate immune response. This result suggests either that disulfiram has broad effects on innate immune cell activities, that formation of NETs in the SARS-CoV-2–infected lungs contributes to further innate immune activation, or — perhaps most likely — a combination of both. Disulfiram treatment also reduced perivascular fibrosis, which would be consistent with reduced perivascular invasion by immune cells and a reduction in associated perivascular edema formation (77) — effects we could not formally measure due to restrictions on experiments involving live SARS-CoV-2. Perivascular fibrosis can lead to increased flow resistance and subsequent pulmonary hypertension, which has been described in patients with severe COVID-19 (78). Perivascular fibrosis is also critical in the context of myocardial damage (79), and gasdermin D inhibition has recently been shown to be protective in acute myocardial infarction (80), so it is possible disulfiram could also ameliorate the cardiovascular symptoms of COVID-19 (46). Disulfiram treatment reduced gene expression of coagulation-related pathways, and it is worth noting that NETs are well known to activate the coagulation cascade and promote thrombi formation (23) and that COVID-19–related coagulopathy is one of the key drivers of mortality (49). Finally, dexamethasone, which is widely used for COVID-19 treatment, appeared to confer less of a benefit on lung pathology in SARS-CoV-2–infected hamsters than disulfiram under our experimental conditions. At the same time, dexamethasone, but not disulfiram, significantly increased the viral load in the lungs when administered from day 1 after infection, consistent with previous reports on the effects of corticoid therapies on respiratory viruses (81). These data support that disulfiram could have utility in the treatment of SARS-CoV-2–infected patients.

Taken together, our data support the notion that disulfiram-mediated blockade of NET formation could be effective at taming the exacerbated immune activation and immunothrombosis seen in severe COVID-19 patients. Additionally, observational studies have suggested a benefit of disulfiram treatment in COVID-19 (82, 83), and clinical trials in the outpatient and inpatient settings are ongoing or recently completed (NCT04485130, https://clinicaltrials.gov/ct2/show/NCT04485130; and NCT04594343, https://clinicaltrials.gov/ct2/show/NCT04594343). Finally, although disulfiram is not specifically a NET inhibitor, its ability to block NETs may be worth exploring beyond COVID-19.

## Methods

### Study design.

The objective of this study was to test the hypothesis that disulfiram can block NET formation and reduce lung injury, including in animal models of TRALI and COVID-19. Sample sizes and endpoints for both the TRALI and COVID-19 models were predetermined by our previous experience with these models. We did not use any data exclusion methods, and no data were excluded. No randomization or blinding methods were used in this study.

### Mice.

All experiments were performed in 7- to 12-week-old, male BALB/c mice (BALB/cAnNCrl) purchased from Charles River Laboratories, housed in a nonbarrier animal facility at CSHL under a 12-hour light/12-hour dark schedule, with water and chow available ad libitum. Mice were acclimatized to the animal housing facility for 1 week prior to performing experiments.

### Flow cytometry and cell sorting.

Flow cytometric analyses were performed using a Fortessa Analyzer (BD Biosciences). Analysis was performed using FlowJo v10 (Tree Star Inc.). Cell sorting experiments were performed using an FACS Aria cell sorter (BD Biosciences). All analyses were conducted at the Flow Cytometry Core Facility at CSHL. The following antibodies were used in this study: CD11b-PE (clone M1/70, Tonbo Biosciences, 50-0112-U100), Ly6G-AF647 (clone 1A8, BioLegend, 127610), and CD45-APC/Cy7 (clone 104, BioLegend, 103116).

Absolute quantification was done using Trucount absolute counting beads (BD Biosciences, 340334) according to the manufacturer’s instructions.

Isolation of blood neutrophils for ex vivo NET formation assays was performed by FACS, as described (84). Briefly, blood was drawn into EDTA-coated tubes, RBCs were lysed in ammonium-chloride-potassium (ACK) lysis buffer (Thermo Fisher Scientific, A1049201), and blood cells were stained with antibodies against Ly6G (BioLegend). Immediately prior to flow cytometric analysis, DAPI was added to the cells so that only viable (DAPI^–^), Ly6G^+^ cells were collected.

Analysis of lung samples by flow cytometry was conducted as previously reported (84). Briefly, the lungs were extracted, placed in cold PBS, and processed immediately afterward. They were digested in HBSS with liberase (1 U/mL, Roche) and DNAse I (1 mU/mL, MilliporeSigma) for 30 minutes at 37°C. Single-cell suspensions were incubated with antibodies against CD45, CD11b, and Ly6G. Immediately prior to flow cytometric analysis, DAPI was added to the suspension.

### Ex vivo NET formation assays with mouse neutrophils.

Neutrophils were sorted as described above, and 4 × 10^4^ neutrophils were plated in serum-free RPMI medium on poly-l-lysine covered 8-well μ-Slides (Ibidi), and left for 30 minutes at 37°C in a cell culture incubator to adhere. Cells were plated in a drop of medium in the center of the well to enhance their adhesion in the central area of the well and to avoid their deposition in the edges. In experiments using RBC-lysed blood, the RBCs were lysed in ACK buffer, the remaining cells were centrifuged (500g at 4°C for 5 minutes), the supernatant was discarded, and the pellet was resuspended directly in RPMI medium. The volume equivalent to 75 μL of the original blood was plated per well, again on poly-L-lysine–covered 8-well μ-Slides as a centered drop and left 30 minutes to adhere as described above. For both sorted neutrophils and RBC-lysed blood, cells were subsequently incubated for 2 hours with 100 nM PMA or vehicle, or PMA together with test compound (at the concentrations indicated in the legends of Figure 1 and Supplemental Figure 1). Cells were then fixed using 4% paraformaldehyde (PFA) in PBS for 10 minutes, blocked, and permeabilized with PBS containing 0.1% Triton X-100, 25% FBS, and 5% BSA; they were stained with antibodies against citrullinated histone 3 (citH3, ab5103, Abcam) and myeloperoxidase (MPO, AF3667, R&D Systems) in 1:200 dilution in blocking buffer, at 4°C overnight. Then, the cells were washed and stained with secondary antibodies: donkey anti–goat-AF647 (A21447, Invitrogen) and donkey anti–rabbit-AF568 (A10042, Invitrogen) 1:400. They were counterstained with DAPI (1:1000) for 2 hours at room temperature. *Z* stack images were acquired with a SP8 Microscope analyzed using Imaris (Bitplane) or using custom-made ImageJ (NIH) macros to identify NETs (defined as triple-positive colocalization events of DNA, citH3, and MPO).

### Antibody-induced ALI.

A 2-event model of TRALI was adopted for our studies, essentially conducted as described (37). Male BALB/c mice from Charles River Laboratories (7–12 weeks old) were injected i.p. with 0.1 mg/kg LPS from *E. coli* (O111:B4, MilliporeSigma). Twenty-four hours later, TRALI was induced by i.v. injecting mice with 1 mg/kg anti-H2d (clone 34-1-2s; Bio X Cell) antibody. Some mice were treated 1 hour before injection of the anti-H2d antibodies (TRALI induction) with 12 mg/kg of Cl-amidine injected i.v. (Cayman Chemical) to block NET formation. Other groups were treated with 200 U of i.n. injected DNase I (DNase I recombinant, Roche) in PBS, 5 minutes before TRALI induction; 0.1 mg/kg tirofiban (tirofiban hydrochloride monohydrate, MilliporeSigma) i.v. injected 1 hour before TRALI induction; 8 mg/kg dipyridamole i.p. injected 24 hours and 3 hours before TRALI induction; 50 μg of anti–IL-1β (InVivoMAb anti–mouse/rat IL-1β, clone B112, Bio X Cell) or isotype control (InVivoMAb polyclonal Armenian hamster IgG, catalog BE0091, Bio X Cell) i.v. injected 5 minutes before TRALI induction; or 50 mg/kg of disulfiram (tetraethylthiuram disulfide from MilliporeSigma) in sesame oil i.p. injected 24 hours and 3 hours before TRALI induction. For survival experiments, mice were observed for 2 hours after TRALI induction (the acute phase of ALI). In some experiments, pO_2_ (%) was measured using a MouseOx Pulse Oximeter (STARR Life Sciences Corp.), approximately every 10 minutes after injection of the anti-H2d antibodies.

### Whole mount immunostaining and tissue clearing.

To determine the abundance of NETs in the lungs of mice after TRALI induction, we performed whole mount immunostaining and tissue clearing of excised lungs as previously described (3). For these experiments, mice were euthanized with CO_2_ 40 minutes after TRALI induction. Mice were then perfused with 20 mL of saline through the left ventricle of the heart, and the lungs were collected in cold PBS. Afterward, lungs were fixed at 4°C overnight in PBS with 4% PFA and 30% sucrose. After 3 washes with PBS for 1 hour each at room temperature, tissues were permeabilized in methanol (MetOH) gradients in PBS (PBS > MetOH 50% > MetOH 80% > MetOH 100%; 30 minutes in each solution). Then, tissues were bleached with Dent’s bleach (15% H_2_O_2_, 16.7% Dimethyl sulfoxide [DMSO] in MetOH) for 1 hour at room temperature and rehydrated through descending methanol gradients in PBS (MetOH 80% > MetOH 50% > PBS, 30 minutes in each solution). Then, tissues were incubated with blocking buffer containing PBS with 0.3% Triton X-100, 0.2% BSA, 5% DMSO, 0.1% azide, and 25% FBS overnight at 4°C with shaking. Afterward, lungs were stained with antibodies against citrullinated histone 3 (rabbit, anti–histone H3-citrulline R2 + R8 + R17 antibody, catalog ab5103, Abcam), MPO (goat, human/mouse myeloperoxidase/MPO antibody, catalog AF3667, R&D Systems), and CD31 (purified rat anti-mouse CD31, clone MEC 13.3, BD Biosciences), all 1:200 in blocking buffer for 2 days at 4°C with shaking. After washing for 24 hours in washing buffer (PBS with 0.2% Triton X-100 and 3% NaCl), the tissues were stained with secondary antibodies donkey anti–rabbit-AF568 (A10042, Invitrogen), donkey anti–rat-AF488 (A212008, Invitrogen), and donkey anti–goat-AF647 (A21447, Invitrogen) 1:400 for 24 hours at 4°C with shaking. Twenty-four hours later, tissues were washed for 24 hours in washing buffer and thereafter dehydrated in MetOH gradients in dH_2_0 using glass containers (MetOH 50% > MetOH 70% > MetOH 90% > 3× MetOH 100%, 30 minutes for each step). Then, tissues were cleared for 30 minutes in 50% MetOH and 50% benzyl alcohol (108006, MilliporeSigma) and benzyl benzoate (BABB, mixed 1:2; B6630, MilliporeSigma), and for 1 hour in 100% BABB; finally, tissues were imaged on an SP8 Microscope (Leica, typical *Z* depths of 200–500 μm). Quantification was performed with Imaris software (Bitplane), using spots on a triple-colocalization channel of DNA, MPO, and citrullinated histone 3. Neutrophils were quantified using spots based on MPO signal. Frequency was calculated as the number of NETs/number of neutrophils in the 3D volume.

### Mouse tissue section immunostaining.

Both 4% PFA-fixed and optimal cutting temperature–embedded (OCT-embedded) tissues were cut in 5 μm sections, incubated for 1 hour at room temperature in blocking buffer (PBS containing 10% BSA and 2% goat serum), and stained for 1 hour at room temperature with 1:200 rabbit anti–mouse laminin (MilliporeSigma) in blocking buffer diluted 1:2 in PBS. Tissues were then washed and further stained for 1 hour at room temperature with Dylight 650–labeled anti-Ly-6G (clone 1A8; Bio X Cell) and phycoerythin-labeled anti-CD41 (eBiosciences) at 1:200, as well as with AlexaFluor 488 goat anti–rabbit IgG (Molecular Probes) diluted 1:500 in blocking buffer. Finally, samples were counterstained with DAPI (MilliporeSigma) and mounted with Mowiol medium (81381, MilliporeSigma). Images were captured on a Leica SP8 microscope.

### CT for lung edema quantification.

Mice were anesthetized with 150 mg/kg ketamine and 10 mg/kg xylazine. Once anesthetized, mice were positioned prone on the imaging cradle and secured with tape and gauze to minimize motion. After a 2D scout scan, a baseline x-ray CT scan covering the lungs and airways was acquired with the CT component of a Mediso nanoScan PET/CT system (Mediso). The x-ray parameters were a beam energy of 50 kVp and exposure of 183 μAs, in an axial scan with 1080 projections. Total scan time was 7 minutes. Images were reconstructed in Nucline software version 3.00.020 (Mediso) to a voxel size of 138 μm isotropic using a Butterworth filter at 100% cutoff. After the baseline scan, the cradle was moved out of the imaging bore so that antibodies against MHC-I could be injected retro-orbitally without disturbing the positioning of the mouse. Immediately following antibody injection, serial CT scans were acquired at 7-minute intervals for up to 49 minutes after injection or until death. Analysis was performed in 3D Slicer (85) (version 4.11.20200930). Briefly, we used the Chest Imaging Platform Extension (revision eefe2ba) to generate the region of interest (ROI) and quantify the mean Hounsfield units (HU) (representing density — i.e., increases in edema content in the airspace are shown as increases in HU) values for the lung. For representation, basal HU values were subtracted from subsequent scans. The 3D volume renders of the lung and edema volume were calculated using Horos v. 3.6.6 (The Horos Project).

### Ex vivo NET formation assay with human neutrophils.

Whole blood was obtained from 3 healthy volunteers (aged 20–40, males and females). The blood was collected by venipuncture into a BD Vacutainer EDTA tube, and RBCs were lysed using ACK lysing buffer (Thermo Fisher Scientific, A1049201). Plating and stimulations were done as for mouse neutrophils.

### IL-1β measurement in the lung.

Lungs were collected and snap-frozen from control mice (treated with low-dose LPS but not injected with anti-H2d antibodies) and from TRALI-induced mice treated with vehicle or disulfiram, 40 minutes after TRALI induction. Pieces of lung tissue (50 mg) were transferred to tubes containing 500 μL of PBS, homogenized at 4°C and centrifuged at 650*g* at 4°C for 5 minutes. Cell-free supernatants were transferred to new tubes and used to determine mouse IL-1β levels by ELISA (R&D Systems, DY401-05) according to the manufacturer’s instructions.

### BALF protein quantification.

For BALF extraction, mice were euthanized in CO_2_ chambers, and the trachea was exteriorized. A silk thread was inserted behind the trachea; then, the trachea was hemisected transversally to allow introduction of a 20 G catheter (Exel Safelet catheter 20G × 1″, Exelint) that was then knotted to the trachea using the silk thread. Then, 1 mmL of saline was introduced into the lungs, carefully recovered, and placed in a sterile 1.5 mL tube on ice. A total of 500 μL of BALF was then centrifuged (300g, 10 minutes, 4°C), and the supernatant was aliquoted and frozen at –80°C. Protein content was measured using the Pierce BCA Protein Assay Kit (Thermo Fisher Scientific) according to the manufacturer’s instructions using 1:10 diluted BALF.

### SARS-CoV-2 propagation, titration, and infection.

SARS-CoV-2 isolate USA-WA1/2020 (NR-52281) was provided by the Center for Disease Control and Prevention and obtained through BEI Resources (NIAID, NIH). SARS-CoV-2 was propagated in Vero E6 cells in DMEM supplemented with 2% FBS, 4.5 g/L D-glucose, 4 mM L-glutamine, 10 mM non-essential amino acids, 1 mM sodium pyruvate, and 10 mM HEPES, and the stock of the virus was prepared using the second passage of the culture. Three days after infection, supernatant containing propagated virus was filtered through an Amicon Ultra 15 (100 kDa) centrifugal filter (MilliporeSigma) at approximately 4000*g* for 20 minutes at 4°C. Flow-through was discarded, and virus was resuspended in DMEM supplemented as described above. Infectious titers of SARS-CoV-2 were determined by plaque assay in Vero E6 cells in Minimum Essential Media supplemented with 2% FBS, 4 mM l-glutamine, 0.2% BSA, 10 mM HEPES, 0.12% NaHCO_3_, and 0.7% agar. All work involving live SARS-CoV-2 was performed in a Centers for Disease Control and Prevention/United States Department of Agriculture–approved (CDC/USDA-ap-proved) BSL-3 facility of the Icahn School of Medicine at Mount Sinai in accordance with institutional biosafety requirements.

### SARS-CoV-2 infections of hamsters.

Three- to 5-week-old male golden hamsters (*Mesocricetus auratus*) were obtained from the Jackson Laboratory. Hamsters were acclimated to the CDC/USDA-approved BSL-3 facility of the Global Health and Emerging Pathogens Institute at the Icahn School of Medicine at Mount Sinai for 2–4 days. All animal procedures were authorized by the Icahn School of Medicine at Mount Sinai. Before intranasal infection, hamsters were anesthetized by i.p. injection with a ketamine HCl/xylazine solution (4:1). Hamsters were i.n. inoculated with 1 × 10^3^ pfu of SARS-CoV-2 in PBS (or PBS only as a control) in a total volume of 100 μL. On the day before infection or the day following infection, and up to the end of the experiments, animals were treated daily by i.p. injection with disulfiram (or vehicle), at a dose of 150 mg/kg in 0.5 mL of sesame oil as vehicle. For dexamethasone treatment, hamsters were s.c. injected daily starting 1 day after infection with 0.2 mg/kg of dexamethasone. In all cases, 6 days after infection, hamsters were euthanized, and lungs were collected (we chose day 6 after infection as it is the time point at which the effects on the lung are most severe, based on our previous experience with the model). For lungs analyzed by immunofluorescence staining, hamsters were perfused with 60 mL of ice-cold PBS before tissue collection, and collected lungs were immediately placed in 10% nonbuffered formalin (NBF) and fixed for 24 hours. For transcriptomic analysis, collected lungs were placed in TRIzol for further RNA extraction.

### RNA-Seq of golden hamster lungs.

Hamster total RNA was extracted in TRIzol (Invitrogen) and DNase I treated using Directzol RNA Miniprep kit (Zymo Research) according to the manufacturer’s instructions. RNA-Seq libraries of polyadenylated RNA were prepared using TruSeq Stranded mRNA Library Prep Kit (Illumina) according to the manufacturer’s instructions. cDNA libraries were sequenced using an Illumina NextSeq 500 platform. The sequencing reads were cleaned by trimming adapter sequences and low-quality bases using cutadapt v1.9.1 30, and they were aligned to the hamster reference genome (downloaded from Ensembl, accession no. GCA 000349665) plus SARS-CoV-2 genome using HISAT2 2.1.0. Raw gene counts were quantified using HTSeq-count v0.11.2. Golden hamster Ensembl genes were matched to homologous external gene names, human homolog Ensembl genes, and human associated homolog gene names using BioMart. OrthoFinder was used to generate orthologous human Ensembl gene identifications and gene names. Differential expression analysis was performed using DESeq2 v1.22.2 33. Regularized log transformation was applied to convert count data to log_2_ scale. Sample-to-sample distance matrix was calculated based on the transformed log-scaled count data using R dist function. Multidimensional scaling (MDS) was performed on the distance matrix using R cmdscale function.

Further analyses were performed using R 4.0.4 (version “Lost Library Book”) and Bioconductor 3.12. Briefly, GO terms were obtained with gProgileR, using a max *P* value of 0.05, and false discovery rate as the correction method. Up- and downregulated gene lists were also analyzed using gProgileR with the same settings. Reactome pathway analysis was performed using the enrichPathway function of the ReactomePA library, using the ENTREZ nomenclature (mapped with the mapIds function of the AnnotationDb library) of the same lists used for the GO terms, with a *q* value and *P* value cutoff of 0.05 in both cases. Only pathways with adjusted *P* value under 0.05 were kept. Volcano plots were represented using the EnhancedVolcano library, with a fold change cutoff of 2 and a *P* value cutoff of 0.05. Reactome pathway clustering was performed using pathfinder: first the run_pathfindR function was run using the same input lists as before, on the Reactome gene set. Then, we used the cluster_enriched_terms function to perform the actual clustering of the Reactome pathways. For KEGG pathways, the procedure was similar, but we used the corresponding gene set. Top genes were extracted with dplyr. SARS-CoV-2 Infection and IFN signaling gene lists were obtained using the viewPathway function of the ReactomePA library. To compare the data sets from hamsters pretreated or treated at the time of infection with SARS-CoV-2, we used the CompGO library. Briefly, functional annotation was acquired using DAVIDWebService by the getFnAnot_genome of the CompGO package for both data sets. Then, the comparison of the *Z* scores was performed using the compareZscores function and *Z* scores correlation plot and sliding Jaccard plots were exported using CompGO. The session used the following libraries: limma (3.46.0), edgeR (3.32.1), tximport (1.18.0), edgeR (3.32.1), sva (3.38.0), RColorBrewer (1.1-2), pheatmap (1.0.12), biomaRt (2.46.3), ggplot2 (3.3.3), gplots (3.1.1), ggfortify (0.4.11), NMF (0.23.0), cluster (2.1.1), fpc (2.2-9), plyr (1.8.6), dplyr (1.0.5), pvclust (2.2-0), ggrepel (0.9.1), amap (0.8-18), gProfileR (0.7.0), xtable (1.8-4), ggpubr (0.4.0), tidyr (1.1.3), DESeq2 (1.30.1), ReactomePA (1.34.0), stringr (1.4.0), Org.Hs.eg.db (3.12.0), pathfindR (1.6.1), CompGO (1.26), EnhancedVolcano (1.8.0), and GeneBook (1.0).

### SARS-CoV-2 nucleocapsid protein quantification in infected lungs.

Standard procedures were followed to perform immunoblotting. Briefly, 90 μg total protein from each lung lysate sample (uninfected control, infected untreated, infected pretreated with disulfiram, infected posttreated with disulfiram) harvested at day 6 after infection was electrophoresed on 4%–20% Mini-PROTEAN TGX Precast Protein Gels (4561094, Bio-Rad) and transferred onto a nitrocellulose membrane using the iBlot 2 Dry Blotting System (IB2001, Invitrogen). Membranes were then blocked with 5% nonfat dry milk in 0.1% Tween-20 Tris-Buffered Saline (TBS-T) and incubated with anti–SARS-CoV-2 nucleocapsid protein primary antibody (Abcam, ab273167, concentration of 2 μg/mL) overnight on a rocking platform at 4°C. Membranes were then incubated with mouse monoclonal (SB62a) anti–rabbit IgG light chain (HRP) (Abcam, ab99697, diluted 1:5,000) for 1 hour at room temperature before proceeding with immunodetection. HRP-conjugated secondaries were visualized by incubating with Radiance ECL (Azure Biosystems, AC2204) for 2 minutes and imaged an Azure 300 Chemiluminescent Western Blot Imaging System (Azure Biosystems). Membranes were then stripped using Restore Western Blot Stripping Buffer (21059, Thermo Fisher Scientific) for 20 minutes at room temperature, washed with TBS-T, and reblocked with 5% nonfat dry milk in 0.1% TBS-T before probing with anti–β-actin (C4) primary antibody (Santa Cruz Biotechnology, sc-47778, diluted 1:500) overnight on a rocking platform at 4°C. Membranes were then incubated with IRDye 800CW near-infrared fluorescent secondary antibody, (LI-COR, 926-32210, diluted 1:10,000) and were detected using an Odyssey Classic Imaging System (LI-COR) and quantified using Image Studio Lite version 5.2.5 (LI-COR). To quantify, background from each image of the blot was subtracted from the signal of the SARS-CoV-2 nucleocapsid (MW: 50 kd) and β-actin (MW: 42 kd) proteins. Then, for each lysate sample run on a single blot (uninfected control, infected untreated, infected pretreated with disulfiram, infected posttreatment with disulfiram), the SARS-CoV-2 nucleocapsid signal was normalized to the β-actin signal of the same lysate.

### Golden hamster lung viral load quantification by real-time PCR.

A single lung lobe was directly homogenized in 1 mL of TRIzol reagent (Invitrogen), and RNA was extracted following the manufacturer’s protocol. In total, 200 ng of RNA was used for one-step RT-qPCR (NEB Luna Universal One-Step RT-qPCR Kit). The reaction mix contained the kit’s master mix, 10 μM of each forward (5′-CTCTTGTAGATCTGTTCTCTAAACGAAC-3′) and reverse primer (5′-GGTCCACCAAACGTAATGCG-3′), 0.5 M betaine, and 10 μg BSA in 10 μL final volume. The cycling conditions were 55°C for 10 minutes, followed by 45 cycles of 95°C for 10 seconds and 60°C for 30 seconds, along with a final melt curve in a Roche light cycler 480 II instrument.

### Analysis of hamster lung slides.

H&E- or Masson trichrome–stained lung slides from disulfiram- or vehicle-treated hamsters were evaluated using QuPath (86) software (version 0.2.3) for quantitative pathology and bioimage analysis. The infiltrated area was calculated using QuPath’s multilayer perceptron artificial neural network (MLP-ANN) pixel classifier trained to the area of highly packed hematoxylin content and compared with total lung area (excluding alveolar space, also calculated using pixel classifiers). The number of neighbors was calculated to generate a cell density map using the QuPath Cell Detection module in H&E-stained slides. To calculate cellularity per area, the total number of cells was compared with total lung area, excluding the alveolar space. Perivascular fibrosis was calculated by measuring the width of the fibrous perivascular tissue normalized to the lumen diameter of the same vessel; we measured 10 individual vessels for each lung section and then averaged them to obtain the mean value per lung. Finally, to quantify neutrophil numbers, unstained sections were deparaffinated, as previously reported (67), and stained for MPO (AF3667, R&D Systems) as stated above. Slides were imaged using a Leica SP8 Microscope, and neutrophil numbers were determined using the Imaris (Bitplane) spots tool and normalized to tissue area.

### Data availability.

We used the hamster reference genome from Ensembl (accession no. GCA 000349665). The SARS-CoV-2–infected golden hamster RNA-Seq has been uploaded to GEO (accession no. GSE180417). All other pieces of data are available upon request. The ImageJ (NIH) macro for ex vivo NET quantification is available in FigShare (DOI: 10.6084/m9.figshare.14401958). RNA-Seq analysis code is available upon request.

### Statistics.

Unless otherwise indicated, data are represented as mean values ± SEM. Paired or unpaired 2-tailed Student’s *t* test was used to compare 2 groups, and more than 2 data sets were compared using 1-way ANOVA with Tukey’s post hoc test. Where applicable, normality was estimated using D’Agostino-Pearson or Shapiro-Wilk normality test. Log-rank (Mantel-Cox) analysis was used for Kaplan-Meier survival curves. The precise tests used are stated in the figure legends. No samples were excluded. All statistical analyses, except for RNA-Seq analysis, were performed using Prism v8 (GraphPad Software). A *P* value below 0.05 was considered statistically significant; nonsignificant differences are indicated in the figures.

### Study approval.

All experiments with mice were conducted in accordance with procedures approved by the IACUC at CSHL and the NIH *Guide for the Care and Use of Laboratory Animals* (National Academies Press, 2011). Whole blood from healthy volunteers was obtained with informed consent and approval by the IRB of CSHL (IRB-13-025). All experiments with hamsters were performed at the Global Health and Emerging Pathogens Institute at the Icahn School of Medicine at Mount Sinai and were authorized by the Icahn School of Medicine at Mount Sinai.

## Author contributions

JMA, LC, JDP, YB, VC, JRM, and MS performed experiments. JMA, SH, and DR performed bioinformatic analyses. JMA, BRT, SKL, RES, and ME designed and supervised experiments. JMA and ME wrote the manuscript, which was edited by all authors.

## Supplementary Material

Supplemental data

Supplemental table 1

Supplemental table 2

Supplemental table 3

Supplemental table 4

Supplemental table 5

Supplemental table 6

Supplemental table 7

Supplemental table 8

Supplemental table 9

Supplemental video 1

Supplemental video 2

Supplemental video 3

Supplemental video 4

Supplemental video 5

## Version 1 02/08/2022

In-Press Preview

## Version 2 03/08/2022

Electronic publication
