# Supplementary material for: Disulfiram inhibits neutrophil extracellular trap formation and protects rodents from acute lung injury and SARS-CoV-2 infection
Source: JCI Insight. 2022 Mar 8;7(5):e157342. doi: 10.1172/jci.insight.157342 (PMC8983145; doi:10.1172/jci.insight.157342)
Supplement: Supplemental data [file jciinsight-7-157342-s270.pdf]

Supplemental material for

Disulfiram inhibits neutrophil extracellular trap formation protecting rodents from acute lung injury and SARS-CoV-2 infection

Jose M. Adrover[1], Lucia Carrau[2], Juliane Daßler-Plenker[1], Yaron Bram[3], Vasuretha Chandar[3], Sean Houghton[4], David Redmond[4], Joseph R. Merrill[1], Margaret Shevik[1,6,7], Benjamin R tenOever[2], Scott Lyons[1], Robert E. Schwartz[3,5,*] and Mikala Egeblad[1,*]

Affiliations:

[1] Cold Spring Harbor Laboratory, Cold Spring Harbor, NY 11724, USA.

[2] Department of Microbiology, Icahn School of Medicine at Mount Sinai. 1468 Madison Ave. New York, NY, 10029, USA.

[3] Division of Gastroenterology and Hepatology, Department of Medicine, Weill Cornell Medicine, 1300 York Ave, New York, NY, 10065, USA.

[4] Division of Regenerative Medicine, Ansary Stem Cell Institute, Weill Cornell Medicine, New York, NY, 10065, USA xx

[5] Department of Physiology, Biophysics and Systems Biology, Weill Cornell Medicine, 1300 York Ave, New York, NY, 10065, USA.

[6] Medical Scientist Training Program, School of Medicine, Stony Brook University, Stony Brook, NY 11794, USA

[7] Graduate Program in Pharmacology, Stony Brook University, Stony Brook, NY 11794, USA

* Correspondence to: Mikala Egeblad, Cold Spring Harbor Laboratory, 1 Bungtown Rd, Cold Spring Harbor, NY 11721, e-mail: egeblad@cshl.edu or Robert E. Schwartz, Weill Cornell Medicine, 413 East 69th Street, New York, NY 10021, e-mail: res2025@med.cornell.edu

Supplemental Figures:

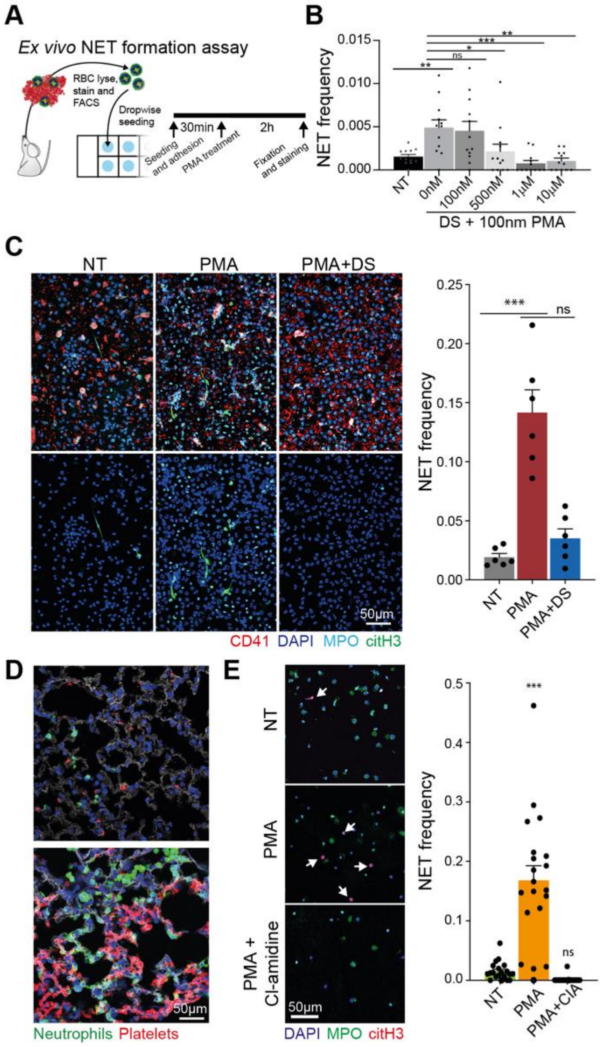

Supplemental Figure 1: Related to Figure 1. (A) Experimental design for *ex vivo* NET formation assay. **(B)** *Ex vivo* NET formation assay using mouse blood neutrophils stimulated with 100nM PMA and increasing doses of disulfiram. **(C)** Images (left) and quantification (right) of the *ex vivo* NET formation assay of red blood cell (RBC)-lysed blood neutrophils unstimulated or stimulated with 100nM of PMA or PMA + 10µM disulfiram (PMA+DS). N = 6 random fields using neutrophils from 3 independent mice per group. The top row of images shows all channels, while the bottom row shows only DAPI and citH3, for clarity. **(D)** Confocal images of lungs from mice treated with LPS only (control, top) or subjected to TRALI induction (bottom) showing neutrophils (Ly6G, green), platelets (CD41, red), laminin (white) and DAPI (blue). **(E)** *Ex vivo* NET formation assay of neutrophils that were unstimulated or stimulated with 100 nM PMA or PMA + 12mg/kg Cl-amidine (PMA+ClA). Arrows mark NETs. Bars show mean ± S.E.M, *P< 0.05, **P< 0.01, ***P< 0.001; n.s., not significant, as determined by one-way ANOVA with Tukey's multiple comparison test.

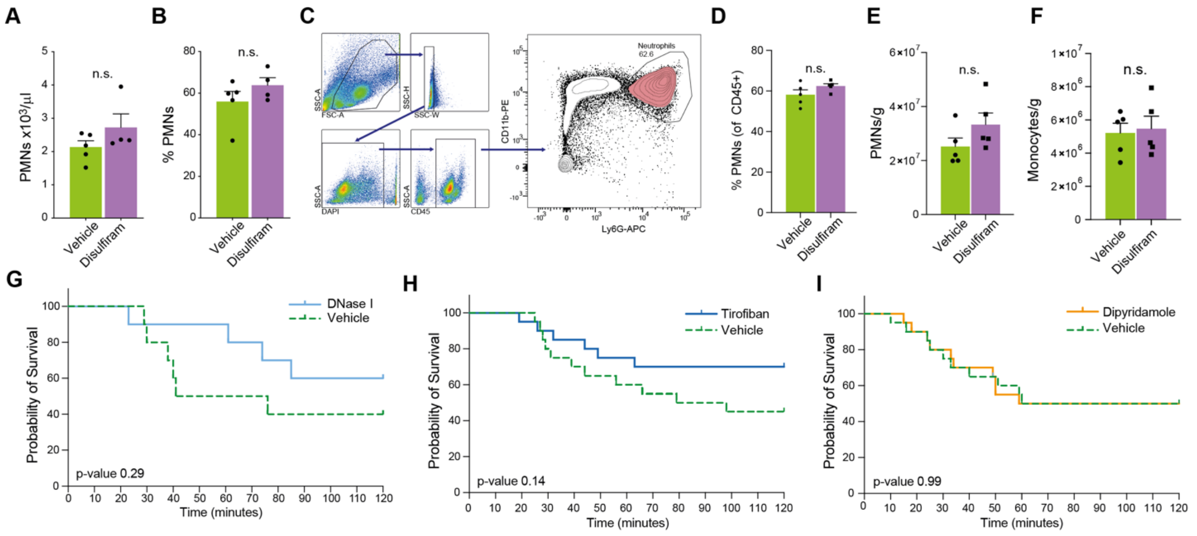

Supplemental Figure 2: Related to Figure 2. (A) Absolute number and **(B)** percentage of neutrophils in the blood of mice treated with disulfiram or vehicle, 40 minutes after TRALI induction. N = 5 vehicle- and 4 disulfiram-treated mice. **(C)** Gating strategy for the lung neutrophil quantification. **(D)** Percentage (of CD45+ leukocytes) and **(E)** Absolute numbers of neutrophils infiltrating the lungs of mice treated with disulfiram or vehicle 40 minutes after TRALI induction. N = 5 mice per group. **(F)** Absolute counts of monocytes infiltrating the lungs of mice treated with disulfiram or vehicle 40 minutes after TRALI induction. N = 5 mice per group. **(G)** Survival curves of mice treated with intranasal DNase I (200U) or vehicle 5 minutes prior to TRALI induction. N = 10 mice per group. **(H)** Survival curves of mice treated with 0.5 mg/kg of tirofiban or vehicle intravenously 1 h before TRALI induction. N = 20 mice per group. **(I)** Survival curves of mice treated intraperitoneally with 8 mg/kg dipyridamole in sesame oil or vehicle 24 and 3 h before TRALI induction, N = 20 mice per group. Bars show mean ± S.E.M, n.s., not significant, as determined by unpaired two-tailed t-test analysis (A, B, D, E, F). Survival curves show probability of survival. Statistics located at the bottom left of each graph determined by log-rank (Mantel–Cox) test (G, H, I).

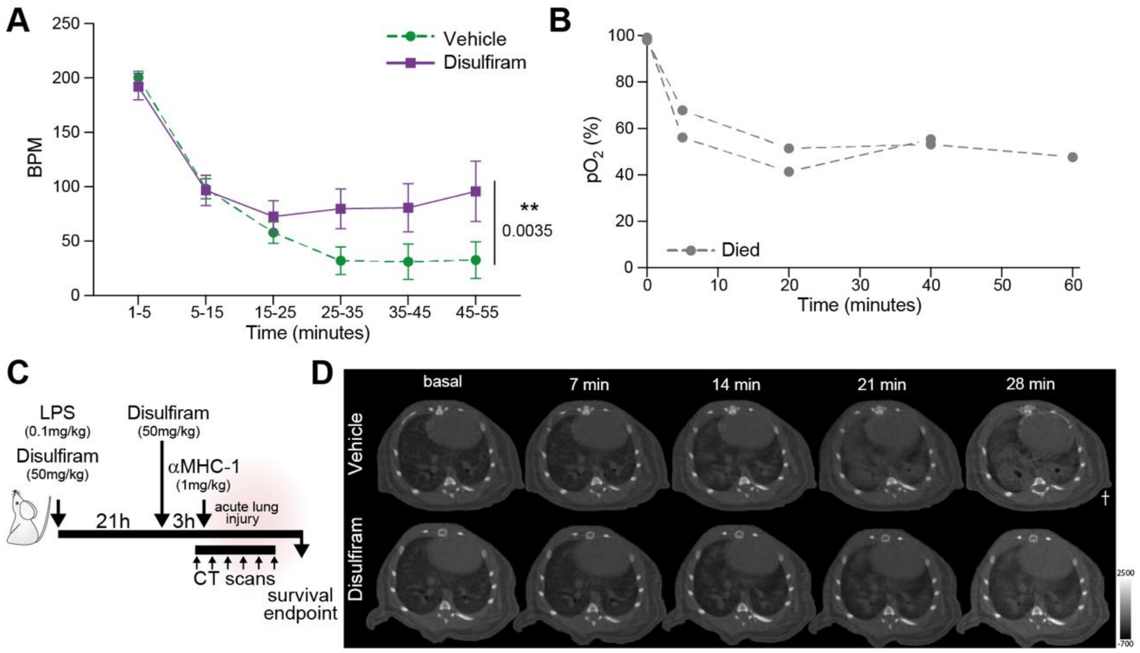

Supplemental Figure 3: Related to Figure 3. (A) Breaths per minute (BPM) over time of mice treated with disulfiram or vehicle after TRALI induction. N = 10 mice per group. **(B)** pO_2 measurement of non-surviving vehicle-treated mice upon TRALI induction. N = 2 mice (each represented by a dashed line). **(C)** Experimental design for the CT scans. **(D)** Representative images of longitudinal CT scans of mice treated with disulfiram or vehicle at indicated time after TRALI induction. Representative of 10 mice per group. Bars show mean ± S.E.M, $^{**}P < 0.01$; as determined by two-way ANOVA (A).

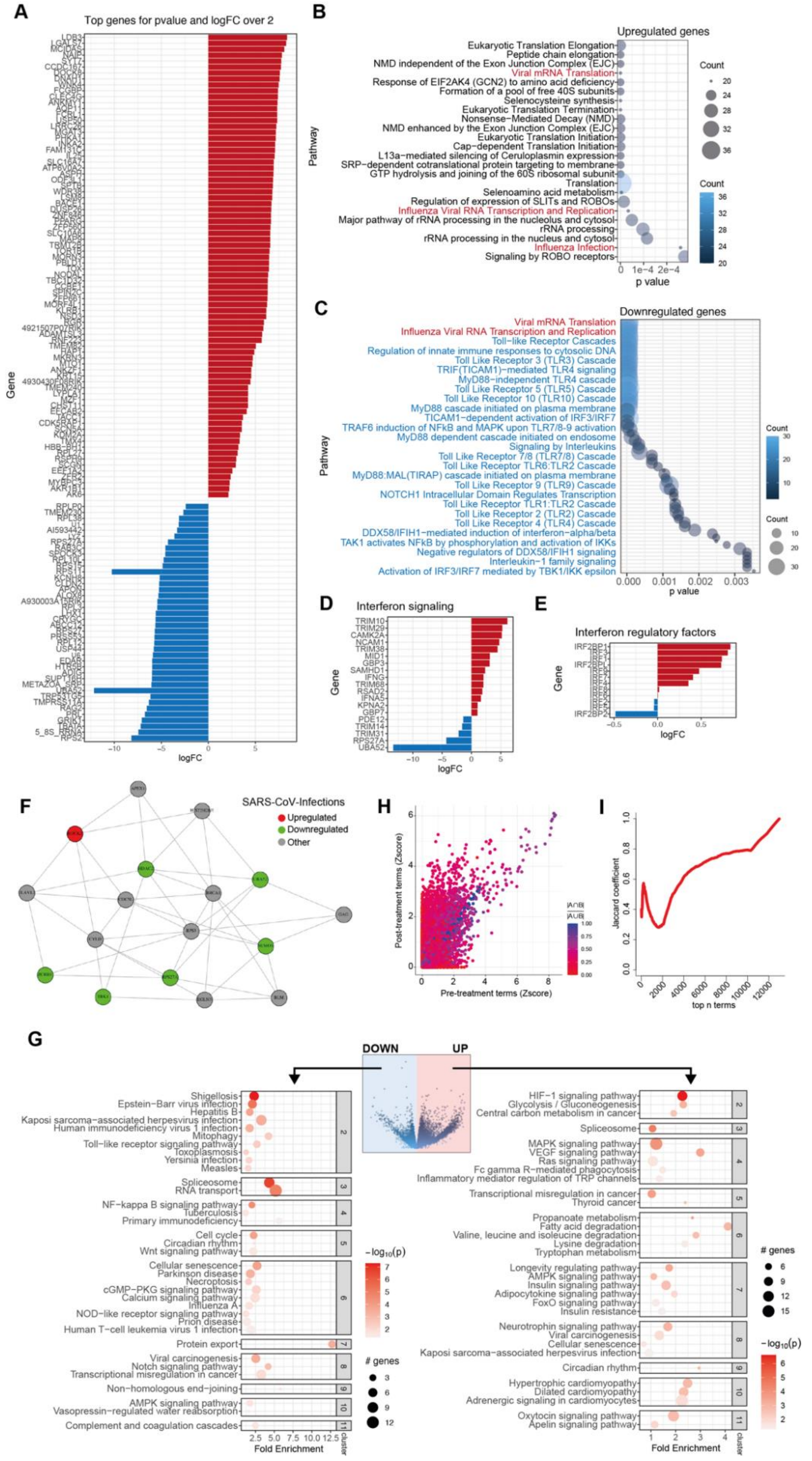

Supplemental Figure 4: Related to Figure 5. (A) Top differentially expressed genes from the RNA-seq of the SARS-CoV-2-infected golden hamsters, showing genes enriched (red)

or repressed (blue) upon disulfiram treatment. **(B)** Reactome pathways enriched in the genes upregulated by disulfiram. **(C)** Reactome pathways enriched in the gene expression downregulated by disulfiram (partial list). **(D)** Overview of interferon signaling genes and **(E)** interferon regulatory factors in the dataset, showing gene expression enriched (red) or repressed (blue) upon disulfiram treatment. **(F)** Overview of the interaction of genes up- and down-regulated by disulfiram of the SARS-CoV-Infections pathway in our dataset. **(G)** Clustering of KEGG pathways enriched in the gene expression downregulated (left) or upregulated (right) in response to disulfiram in SARS-CoV-2-infected golden hamsters. **(H)** Scatterplot comparing the GO terms of the disulfiram pre- and post-treatments by p-value showing their similarity (Jaccard index, $|A \cap B| / |A \cup B|$). **(I)** Comparison of pre- and post-treatment functional annotation using a sliding-jaccard coefficient, showing a high overlap in the first 200 terms.

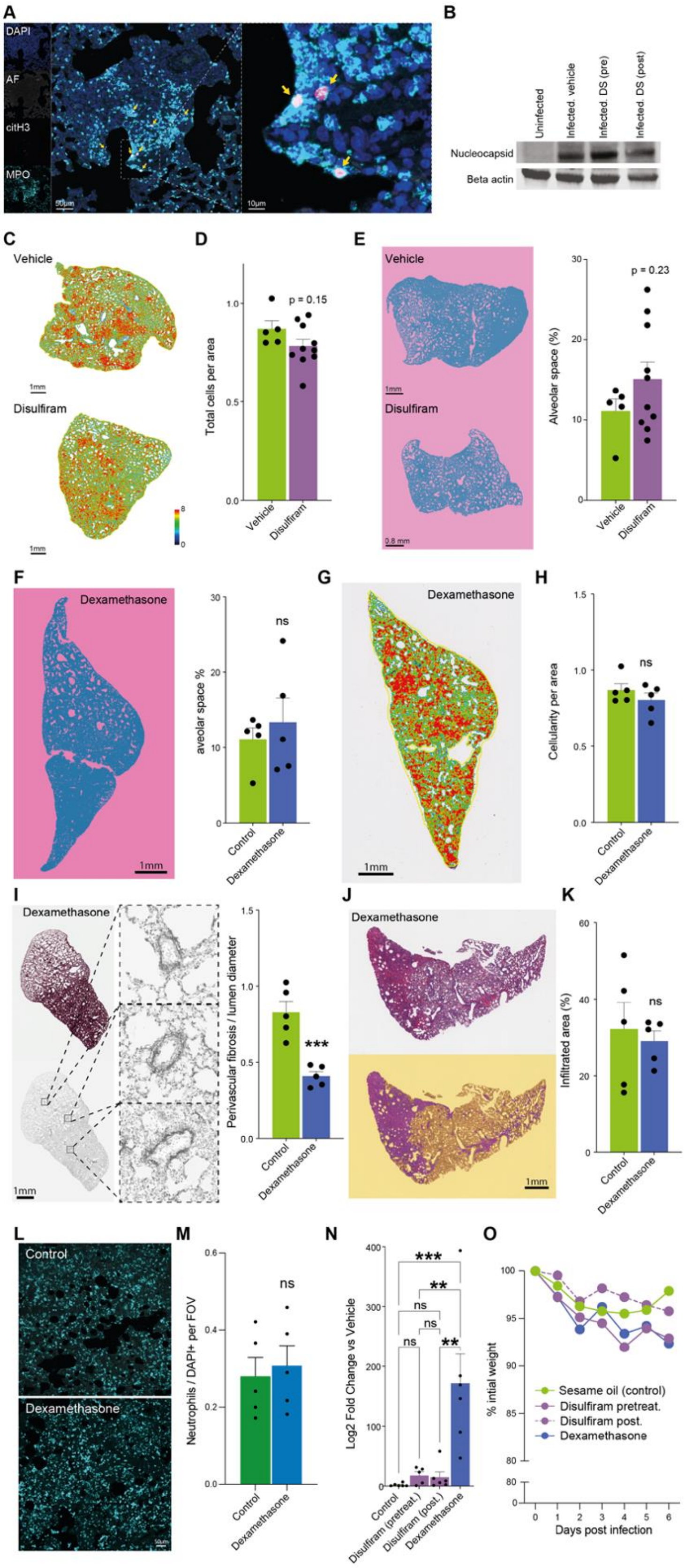

Supplemental Figure 5: Related to Figure 4. (A) Representative image of NETs in the lungs of SARS-CoV-2-infected golden hamsters. Representative of lungs from five hamsters.

AF: auto-fluorescence. Yellow arrows point to NETs. **(B)** Representative Western blot of nucleocapsid protein and beta-actin in lung lysates from the hamsters. Representative of 5 hamsters per group, except uninfected, where N = 3. **(C)** Density map (left, showing number of neighbors per cell) and **(D)** quantification of the number of cells per area in H&E-stained sections of SARS-CoV-2-infected hamsters treated with disulfiram or vehicle. N = 5 (vehicle) and 10 (disulfiram) hamsters per group. **(E)** Representative segmented images (left) and quantification (right) of open alveolar space from H&E-stained sections of SARS-CoV-2-infected hamsters treated with disulfiram or vehicle. N = 5 (vehicle) and 10 (disulfiram) hamsters per group. **(F)** Representative segmented images (left) and quantification (right) of open alveolar space from H&E-stained sections of SARS-CoV-2-infected hamsters treated with dexamethasone. N = 5 hamsters per group (controls as in panel e for reference. Note that dexamethasone treated hamsters were infected and treated at the same time as disulfiram treated hamsters). **(G)** Density map (showing number of neighbors per cell) and **(H)** quantification of the number of cells per area in H&E-stained sections of SARS-CoV-2-infected hamsters treated with dexamethasone. N = 5 hamsters per group (controls as in panel d for reference). **(I)** Representative images (left) and quantification (right) of perivascular fibrosis in the Masson trichrome-stained lungs of infected hamsters treated with dexamethasone. N = 5 hamsters per group. **(J)** Representative images and **(K)** quantification of the heavily immune-infiltrated areas from hematoxylin and eosin-stained lungs of dexamethasone-treated hamsters infected with SARS-CoV-2 (controls as in Figure 4g for reference). N = 5 lungs per group. **(L)** Representative images (showing MPO signal in cyan) and **(M)** quantification of neutrophil infiltration to the lungs of SARS-CoV-2-infected golden hamsters treated with Dexamethasone (controls as in Figure 4d for reference). N = 30 random fields from 5 lungs per group. **(N)** Viral load quantification by real-time PCR from lungs of hamsters treated with vehicle (control), disulfiram (pre- and post-infection treatment groups) and dexamethasone. N = 6 hamsters (vehicle, disulfiram post-treatment and dexamethasone groups) and 5 hamsters (disulfiram pre-treatment group). **(O)** Weight loss curves of all groups, showing no differences between all groups. N = 6 hamsters per group. Bars show mean ± S.E.M, ***P< 0.001; n.s., not significant and p-values shown as determined by unpaired two-tailed Student's t-test analysis (D, E, F, H, I, K, M) or one-way ANOVA with Tukey's multiple comparison test (N).

Legends for supplemental videos:

- **Supplemental Video 1: NETs are abundant in the lungs of TRALI-induced mice treated with vehicle.** Representative whole mount tissue clearing of lungs from vehicle-treated mice subject to TRALI, and stained for the vasculature (CD31, grey), DNA (DAPI, blue), neutrophils (MPO, cyan) and citrullinated histone 3 (citH3, red).
- **Supplemental Video 2: NETs are scarce in the lungs of TRALI-induced mice treated with disulfiram.** Representative whole mount tissue clearing of lungs from Disulfiram-treated mice subject to TRALI, and stained for the vasculature (CD31, grey), DNA (DAPI, blue), neutrophils (MPO, cyan) and citrullinated histone 3 (citH3, red).
- **Supplemental Video 3: Disulfiram-treatment reduces edema formation in the lungs of mice subject to TRALI.** 3D reconstructions of CT scans showing the bone, lung and edema volumes in mice treated with Disulfiram or vehicle, at baseline (before TRALI induction) and 21 minutes after TRALI induction.
- **Supplemental Video 4: NETs are abundant in the lungs of SARS-CoV-2-infected golden hamsters treated with vehicle.** Representative whole mount tissue clearing of lungs from vehicle-treated golden hamsters infected with SARS-CoV-2, 6 days post-infection, and stained for the DNA (DAPI, blue), neutrophils (MPO, cyan) and citrullinated histone 3 (citH3, red). Autofluorescence is shown in gray for reference.
- **Supplemental Video 5: NETs are scarce in the lungs of SARS-CoV-2-infected golden hamsters treated with disulfiram.** Representative whole mount tissue clearing of lungs from Disulfiram-treated golden hamsters infected with SARS-CoV-2, 6 days post-infection, and stained for the DNA (DAPI, blue), neutrophils (MPO, cyan) and citrullinated histone 3 (citH3, red). Autofluorescence is shown in gray for reference.

Legends for supplemental tables

- **Supplemental Table 1:** Differential expression analysis of the RNA-seq dataset comparing disulfiram- vs. vehicle-treated lungs (treated 24 h prior to infection) from SARS-CoV-2-infected hamsters.
- **Supplemental Table 2:** GO terms (biological processes) of the differentially expressed genes comparing disulfiram- vs. vehicle-treated lungs from SARS-CoV-2-infected hamsters.
- **Supplemental Table 3:** Reactome pathway analysis of the genes downregulated in response to disulfiram in SARS-CoV-2-infected hamsters.
- **Supplemental Table 4:** Clustering of the Reactome pathway analysis of the genes downregulated in response to disulfiram in SARS-CoV-2-infected hamsters.
- **Supplemental Table 5:** Clustering of the Reactome pathway analysis of the genes upregulated in response to disulfiram in SARS-CoV-2-infected hamsters.
- **Supplemental Table 6:** Differential expression analysis of the RNA-seq dataset comparing disulfiram- vs. vehicle-treated lungs (treatment started one day after infection) from SARS-CoV-2-infected hamsters.
- **Supplemental Table 7:** Reactome pathway analysis of the differentially expressed genes comparing disulfiram- vs. vehicle-treated lungs (treatment started one day after infection) from SARS-CoV-2-infected hamsters.
- **Supplemental Table 8:** GO terms (biological processes) of the genes downregulated in response to disulfiram (treated one day after infection) in SARS-CoV-2-infected hamsters.
- **Supplemental Table 9:** Common GO terms on both treated 24 h prior to infection and treated one day after infection lung RNA-seq datasets (defined as not significantly different).
